# Fast and High‐Resolution luminal water imaging for prostate cancer diagnosis

**DOI:** 10.1002/mrm.30628

**Published:** 2025-07-04

**Authors:** Hao Li, Nikita Sushentsev, Dimitri Kessler, Shaohang Li, Kang‐Lung Lee, Andrew Nicholas Priest, Ferdia A. Gallagher, Tristan Barrett

**Affiliations:** ^1^ Institute of Science and Technology for Brain‐Inspired Intelligence Fudan University Shanghai People's Republic of China; ^2^ Department of Radiology University of Cambridge Cambridge United Kingdom; ^3^ Department of Radiology Taipei Veterans General Hospital Taipei Taiwan; ^4^ School of Medicine National Yang Ming Chiao Tung University Taipei Taiwan; ^5^ Department of Radiology Cambridge University Hospitals NHS Foundation Trust, Addenbrooke's Hospital Cambridge United Kingdom

**Keywords:** imaging acceleration, luminal water imaging, multicomponent T_2_, multi‐echo spin‐echo, prostate MRI

## Abstract

**Purpose:**

This study aims to address the limitation of long acquisition times in luminal water imaging (LWI), a promising noninvasive MRI technique for prostate cancer detection and grading, by implementing an accelerated multi‐echo spin‐echo method termed T2 mapping using echo merging plus k‐t undersampling with reduced flip angles (*TEMPURA)*.

**Methods:**

TEMPURA enables faster acquisition through echo merging, *k*‐t undersampling, and reduced refocusing flip angles. A prospective study was conducted on 24 patients (age 59–75 years) with biopsy‐proven prostate cancer. Imaging was performed on a 3 T MRI system, comparing two TEMPURA‐based LWI sequences—Fast (standard resolution) and High‐Resolution (HR, doubled spatial resolution)—against a standard LWI sequence (Standard). Luminal water fraction and five additional parameters were compared across the methods. Statistical analyses included Spearman rank correlation, Wilcoxon rank sum test, receiver operating characteristic analysis, Bland–Altman plots, and Delong tests.

**Results:**

Compared to Standard, Fast reduced the acquisition time from 8.3 to 2.8 min, whereas HR reduced it to 5.4 min and doubled spatial resolution. Both Fast and HR showed high correlation with Standard for luminal water fraction (*r* = 0.97/0.90 in peripheral zone, *r* = 0.91/0.93 in transition zone), with low bias (0.014/0.018 in peripheral zone, 0.024/0.024 in transition zone) and no significant differences (*p* = 0.05–0.84/0.08–0.51). No significant difference was observed in area under the curve values between Fast/HR and Standard among all parameters (*p* = 0.05–0.87).

**Conclusion:**

The acceleration method greatly reduced the acquisition time and increased the spatial resolution of LWI. Compared with the Standard acquisition, both the Fast and HR methods showed a high correlation for LWI measurements and consistent diagnostic performance in detecting malignant lesions.

## 1 INTRODUCTION

Although multiparametric MRI is widely utilized for prostate cancer (PCa) diagnostics, its efficacy in accurately assessing aggressiveness is not well established. The current gold standard for PCa grading relies on invasive biopsy sampling and Gleason scoring, highlighting the need for noninvasive techniques to improve grade determination.

Advanced models based on the multi‐compartmental structure of prostate tissue provide detailed histological estimates, offering a comprehensive understanding of underlying tissue.^1^, ^2^, ^3^ Alongside diffusion imaging models, luminal water imaging (LWI) has emerged as a promising T_2_‐based approach for PCa detection^4^, ^5^ and grading.^6^ LWI obtains multiple parameters from a series of consecutive T_2_‐weighted images and uses multi‐exponential modeling to differentiate T_2_ values within prostate tissue compartments. This facilitates a noninvasive assessment of luminal water content,^7^ a parameter that can significantly change with disease progression.

However, LWI relies on the time‐consuming multi‐echo spin‐echo (MESE) sequence, requiring long TEs (900–1600 ms)^4^, ^5^, ^6^, ^7^, ^8^, ^9^, ^10^ for detecting the luminal water signal. Conventional MESE‐based LWI used previously^4^, ^6^, ^7^, ^8^, ^9^ required over 8 min for the acquisition of 12 slices with relatively low resolution (approximately 2 × 2 mm^2^). Efforts to shorten acquisition times include faster sequences such as inner‐volume selection– gradient and spin‐echo (GRASE) FOCal Underdetermined System Solver^10^ and *k*‐space undersampling using parallel imaging and/or compressed sensing, specifically in both the spatial and temporal (echo) dimensions.^11^, ^12^, ^13^, ^14^, ^15^, ^16^ The time efficiency of MESE can also be improved by combining adjacent echoes into a single *k*‐space,^17^, ^18^, ^19^, ^20^ but this strategy has been limited to conventional T_2_‐mapping and has not been combined with compressed sensing undersampling.

We have recently developed a highly accelerated MESE‐based approach for renal T_2_ measurement: T_2_ mapping using echo merging plus *k*‐t undersampling with reduced flip angles (TEMPURA).^21^, ^22^ It combines adjacent echoes into a shared *k*‐space and applies *k*‐t compressed sensing reconstruction and extended phase graph (EPG)–based fitting under a reduced flip angle scheme. Nevertheless, the limited TEs and relatively small number of echoes in conventional T_2_‐mapping restrict the potential for high acceleration along the echo dimension. In contrast, LWI, with its particularly long T_2_ values, poses challenges in scan duration but also offers greater opportunities for acceleration through k‐t undersampling and echo combination.

This study applies TEMPURA to prostate LWI, developing Fast and HR methods evaluated in patients with biopsy‐proven PCa. Their performance was compared with a Standard LWI (Standard) acquisition.^8^

## 2 METHODS

### 2.1 Acquisition schemes

**FIGURE 1:**
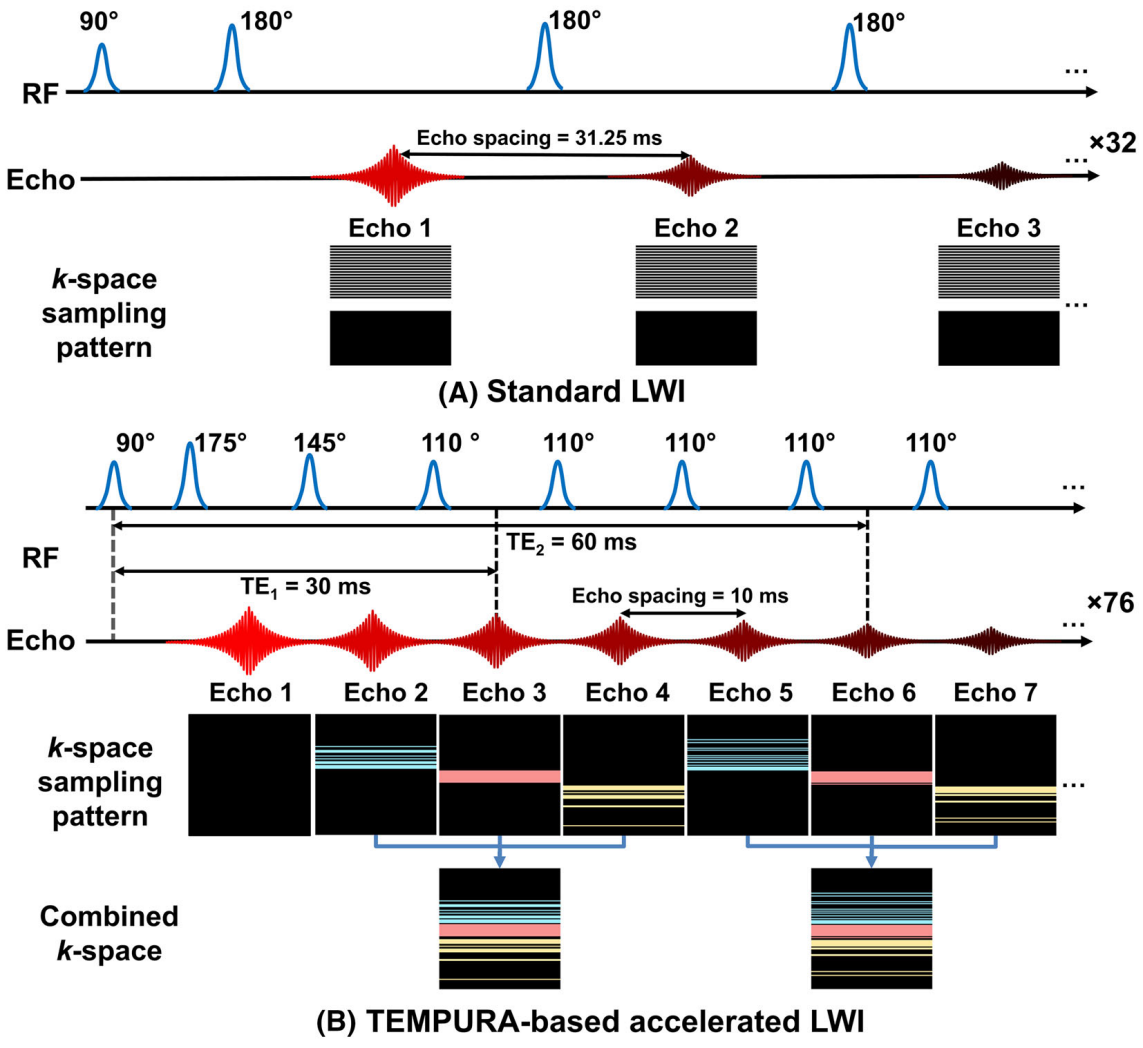
Schematic diagram of the multi‐echo spin‐echo sequences for LWI. (A) Standard LWI using constant flip angles with SENSE (×2) and half‐Fourier sampling. (B) Accelerated LWI based on TEMPURA. LWI, luminal water imaging; TEMPURA, T_2_ mapping using echo merging plus *k*‐t undersampling with reduced flip angles.

The acquisition schemes of Standard and TEMPURA‐based accelerated LWI are shown in Figure 1. Standard uses constant 180° flip angles and a large echo spacing of 31.25 ms, with acceleration by SENSE (×2) and half‐Fourier sampling.^8^ TEMPURA combines every three consecutive echoes into one *k*‐space, randomly undersampled at ×2.5, achieving a total acceleration factor of 7.5. Refocusing flip angles are reduced (175°–145°–110°…–110°) to lower the specific absorption rate, and echo spacing is shortened to 10 ms, allowing more echoes for compressed sensing reconstruction. The first echo is discarded to minimize stimulated echo effects.

Two LWI protocols were established based on TEMPURA: a fast one (Fast) maintaining the same pixel size as Standard, and a High‐Resolution (HR) one featuring a halved pixel size. Both protocols demonstrate shorter acquisition times compared to Standard. Key parameters are detailed in Table 1.

**TABLE 1:** Key parameters of luminal water imaging sequences

Method	Acceleration	TE (min:Δ:max)	TR (ms)	Flip angle	Pixel size (mm)	Bandwidth (Hz/pixel)	Time (min:s)
Standard	SENSE: 2× Half‐Fourier: 1.6×	31.25:31.25: 1000	6091	180°	1.88 × 1.88	244	8:20
Fast	Echo‐comb: 3× Undersampling: 2.5×	30:30:760	4638	110°	1.88 × 1.88	976	2:48
HR	Echo‐comb: 3× Undersampling: 2.5×	30:30:760	4638	110°	0.94 × 0.94	326	5:25

Other common parameters for all three approaches: FOV = 240 mm, 12 slices with thickness/gap of 4.2/0.8 mm. Echo‐comb, Echo‐combination; HR, High‐Resolution; Time, acquisition time.

### 2.2 Reconstruction and fitting

The combined k‐space was reconstructed using a *k*‐t method based on *k*‐t FOCal Underdetermined System Solver (FOCUSS),^23^ incorporating principal component analysis as the sparsifying transform.^11^ Further details are presented in Ref. ^21^.

LWI parameters were calculated with the DECAES toolbox based on the Julia programming language.^24^ DECAES calculates voxelwise T_2_ distributions using regularized nonnegative least‐squares inversion, and the EPG^25^, ^26^ was incorporated to correct for stimulated echoes caused by reduced refocusing flip angles. A fixed T_1_ of 1500 ms and ideal B_1_ were used in all processing, consistent with prior work,^21^ because the EPG model and LWI parameters are relatively insensitive to T_1_ and B_1_ variations. Six parameters were generated from the T_2_ distributions, including luminal water fraction (LWF), geometric mean T_2_, geometric mean of the short (T_2‐short_) and long (T_2‐long_) components, and areas under the short (A_short_) and long (A_long_) components.^4^ All other reconstruction and processing were performed using MatLab 2023a (MathWorks, Natick MA).

### 2.3 MRI data acquisition

In vivo data were acquired prospectively from 24 patients with biopsy‐proven prostate cancer who were enrolled between December 2021 and December 2022. Participating patients were on active surveillance and had no prior treatment for PCa or contraindications to MRI. The study was approved by the local research ethics boards, and all patients provided written informed consent.

Imaging was performed using a 3 T system (Discovery MR750; GE Healthcare, Waukesha, WI) and a 32‐channel cardiac array coil. All patients underwent scanning with Standard, Fast, and HR LWI. Other sequences in a routine protocol include axial fast spin‐echo T_1_W image, axial fast spin‐echo T_2_W image, and axial DWI (*b*‐values: 100, 750, 1400 s/mm^2^) for anatomical reference and standard‐of‐care clinical interpretation.

### 2.4 Data processing

Regions of interest (ROIs) were drawn manually by two urogenital radiologists (with 3 years of experience and 6 years of experience), supervised by another experienced radiologist (with 14 years of experience), in consensus using open‐source segmentation software ITK‐SNAP (Penn Image Computing and Science Laboratory, Philadelphia, PA). Tumor ROIs included Prostate Imaging Reporting and Data System v2^27^ scores ≥3 lesions that harbored biopsy‐proven PCa. ROIs were drawn to include all tumor regions with an average as the value for one tumor. Extratumoral ROIs were drawn to include regions of normal‐appearing prostate that were proven to be benign on biopsy. At least three ROIs on different slices were drawn for benign tissues, with the average value calculated accordingly. ROIs were placed in transition zone (TZ) and peripheral zone (PZ) separately on T_2_W images and then converted to registered LWI maps.

### 2.5 Statistical analysis

Spearman rank correlation coefficients were calculated to assess the correlation of Fast/HR with Standard measurements. Receiver operating characteristic (ROC) analysis based on a logistic generalized linear model was performed to assess the diagnostic performance of LWI parameters obtained by different methods for differentiation between benign tissues and malignant lesions. Area under the curve (AUC) was calculated and compared between Fast/HR and Standard using a Delong test. Wilcoxon rank sum test was used to determine significant differences in LWI parameters between Standard and Fast/HR methods. *P*‐values <0.05 were considered statistically significant for all analyses. Statistical analysis was performed using MathLab 2023a (MathWorks).

## 3 RESULTS

The median age of the 24 included patients was 69 years (interquartile range: 59–75 years), and the median prostate‐specific antigen was 6.23 ng/mL (range 2.49–14.86 ng/mL). Table S1 displays the Gleason and PI‐RADS scores of the 29 index lesions identified among the enrolled patients. The median time from biopsy to MRI was 334.5 days (interquartile range 286–662.5). In addition, six of the 24 patients had subsequent repeat biopsy events, with no change in pathology.

A sample size calculation was performed to determine the number of patients required for the detection study. Based on an estimated effect size and desired statistical power of 0.80, a minimum of 24 patients was deemed sufficient to detect significant differences in LWI parameters between benign and malignant tissues.

**FIGURE 2:**
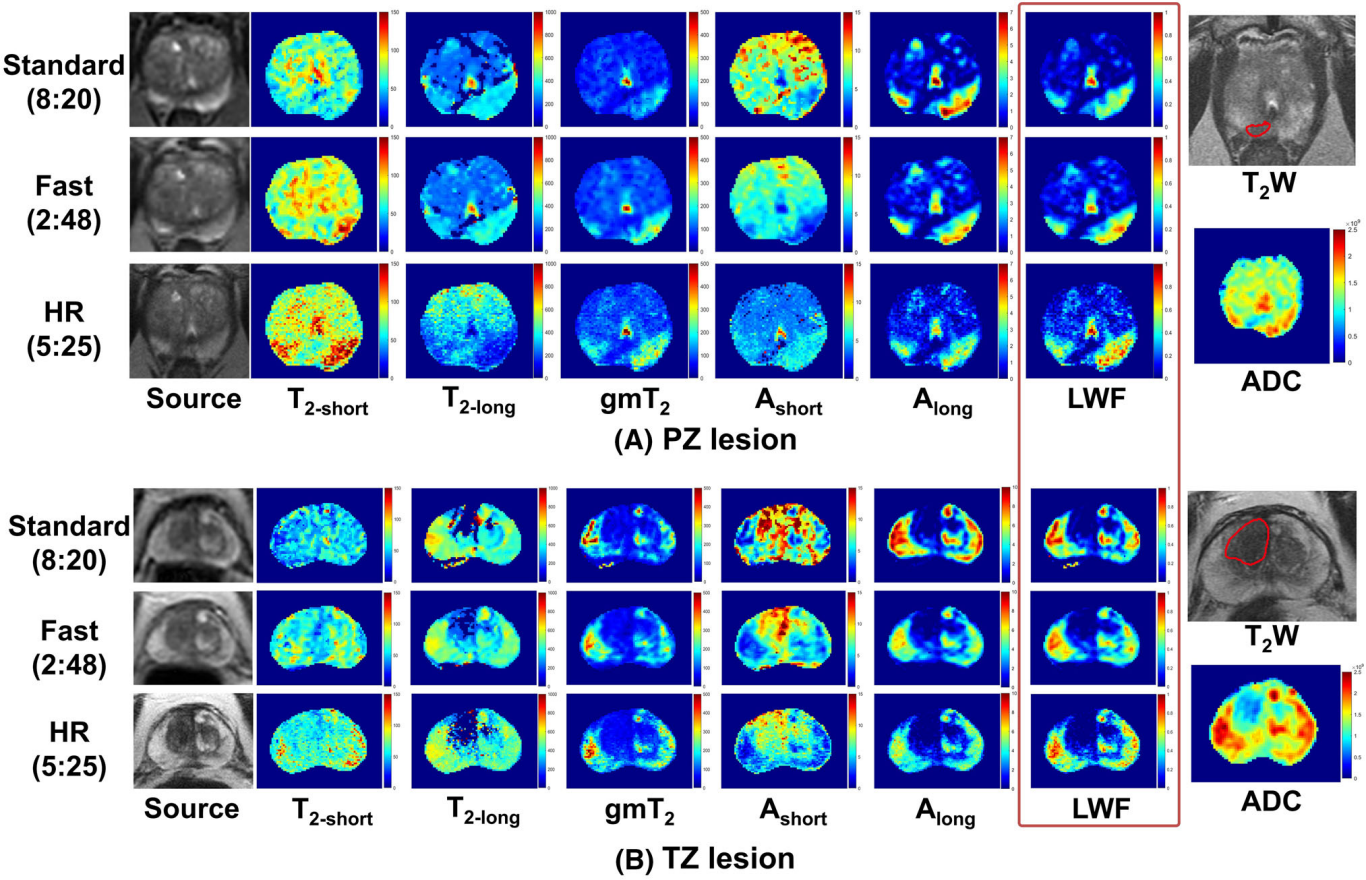
Source T_2_W images from LWI (TE = 90 ms for Fast/HR and 93.75 ms for standard), representative maps of LWI parameters, separately acquired T_2_W (TE = 103.5 ms), and ADC map from two patients with histologically confirmed malignant lesions in PZ (A) and TZ (B). The red outlines on the T_2_W images indicate the lesions. HR, High‐Resolution; PZ, peripheral zone; T_2_W, T_2_ weighted; TZ, transition zone.

Figure 2 shows representative maps of LWI parameters from two patients with histologically confirmed malignant lesions in TZ and PZ, respectively. Both Fast and HR techniques generated LWI maps with a similar appearance but reduced acquisition times. HR provided more precise details and clearer tumor boundaries. Tumors can be identified as a low signal‐intensity region on the LWF maps using all three methods, which aligns with the manually drawn, biopsy‐proven ROI and the low signal‐intensity region on the ADC map.

### 3.1 LWI parameter quantification

Quantitative average measurements of malignant and benign tissues from all patients are summarized in Table S2. Both Fast and HR showed significant correlation with Standard on all parameters except T_2‐short_ PZ for HR. Fast and HR both showed a high correlation coefficient (0.97/0.90 in PZ and 0.91/0.93 in TZ) and low bias (0.014/0.018 in PZ and 0.024/0.024 in TZ) to Standard in LWF measurements. Although significant differences exist in some intermediate parameters, there was no significant difference in LWF between Standard and Fast/HR (*p* = 0.37/0.51, 0.84/0.44, 0.05/0.19, 0.16/0.08 for malignant PZ, benign PZ, malignant TZ, and benign TZ, respectively).

**FIGURE 3:**
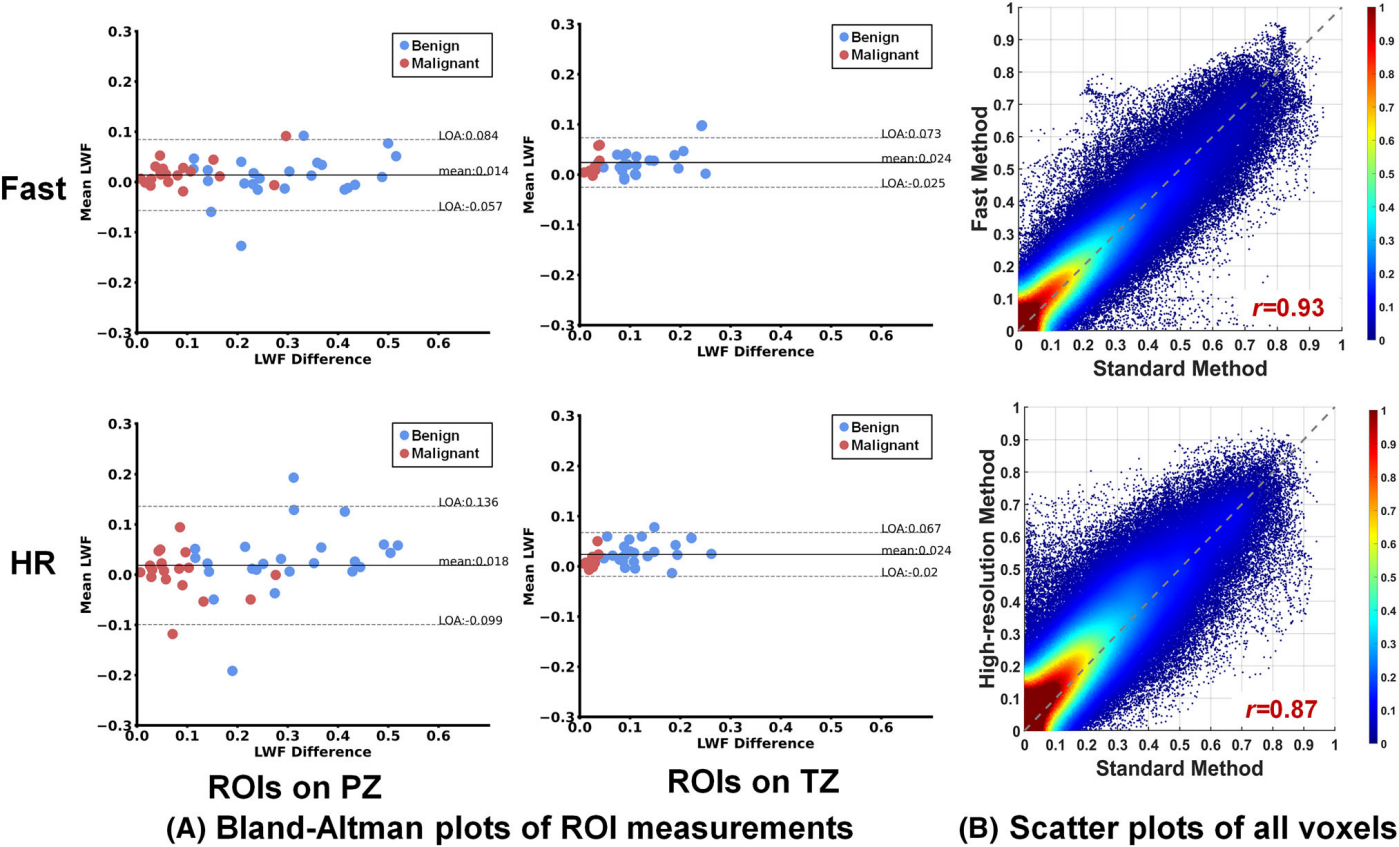
Comparison of LWF for different methods. (A) Bland–Altman plots of ROI measurements from all patients and (B) scatter plots with normalized density for all prostate voxels on LWF maps from five randomly selected patients. LWF, luminal water fraction; ROI, region of interest.

The consistency in LWF can also be observed in Figure 3, showing Bland–Altman plots of ROI measurements from all patients and scatter plots of all prostate voxels in five randomly selected patients.

### 3.2 Diagnostic Performance

**FIGURE 4:**
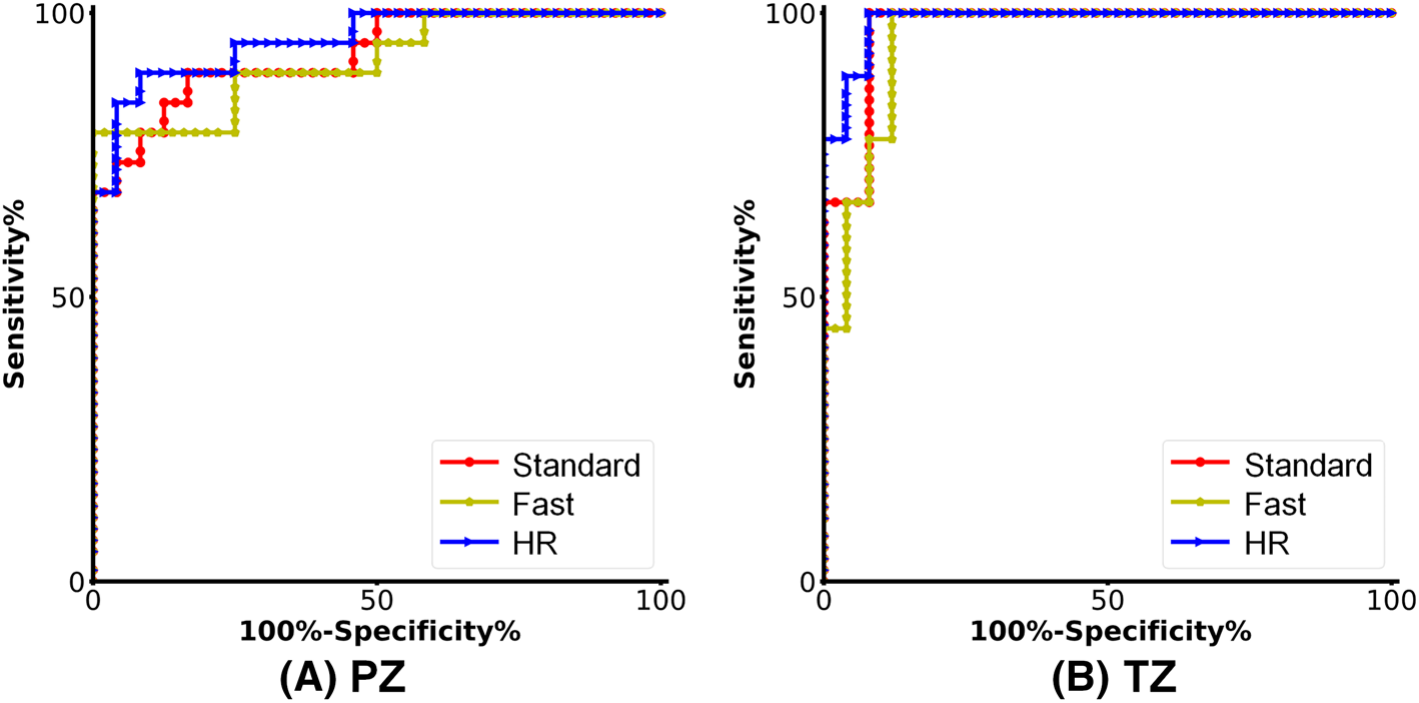
ROC curve analysis of malignant lesions in PZ (A) and TZ (B) based on LWF for the Standard, Fast, and HR methods. ROC, receiver operating characteristic.

Table S3 shows ROC analysis for detecting PCa with a Delong test for statistical analysis, and Figure 4 shows the corresponding ROC curves of LWF. No significant difference exists in diagnostic performance between Fast/HR and Standard among all parameters. The LWF values acquired by the three methods all demonstrated high AUC values in both PZ and TZ (0.92–0.99), with HR showing the highest values (0.95 for PZ and 0.99 for TZ), although not significantly (DeLong *p*‐value 0.332 and 0.545).

## 4 DISCUSSION

In this study, a rapid (Fast) and a high‐spatial‐resolution (HR) LWI sequence were both developed based on TEMUPRA. The results show that the Fast sequence reduced the acquisition time by threefold (from 500 to 168 s), and HR reduced the acquisition time by half and doubled resolution in both in‐plane directions (from 1.88 × 1.88 to 0.94 × 0.94 mm^2^). Compared to Standard, both Fast and HR methods demonstrated high correlation in most LWI measurements and no significant difference in the diagnostic performance. The Fast sequence may therefore be useful for the diagnosis of PCa requiring quick examinations, whereas the HR sequence may provide distinct depiction of tumor boundaries and enhanced visualization of anatomical structures.

TEMPURA has previously been applied to renal T_2_ mapping, enabling a Fast acquisition in one breath‐hold.^21^ Compared to conventional T_2_ mapping using a limited number of echoes, LWI is better suited for TEMPURA because it has a much longer temporal (echo) dimension that can facilitate echo merging and undersampling. Previous MESE‐based LWI studies typically used a maximum TE of 900–1600 ms, with 30–64 echoes and an echo spacing of 31.25 or 25 ms.^4^, ^6^, ^7^, ^8^, ^9^ TEMUPRA reduces the echo spacing to 10 ms; therefore, more echoes (76 echoes in this study) can be acquired and then be merged to reduce the acquisition time. In addition, reduced flip angles (110°) are used to reduce the energy deposition resulting from refocusing pulses. The effects of these reduced flip angles on the echo amplitudes are compensated by fitting to an extended phase graph model.

Bias and low‐correlation coefficients can be observed in intermediate parameters such as T_2‐long_, T_2‐short_, A_short_, and A_long_. This might be attributed to the stimulated echo effect or echo combination, resulting in varying specific T_2_ distributions. Despite this, the diagnostic accuracy of Fast and HR using these parameters, as measured by AUC, did not decrease. Notably, there was no significant difference observed between Fast/HR and Standard for the most significant biomarker LWF,^4^, ^7^ which is defined by the area fraction of the longer T_2_ component.

Similar to TEMPURA, the GRASE sequence also utilizes echo sharing and has been applied to LWI to reduce the acquisition time.^5^, ^10^ Nonetheless, the GRASE sequence faces limitations such as underestimation of T_2_ due to introducing T_2_* weighting,^28^ image blurring induced by the lower SNR gradient echoes, and more serious peripheral nerve stimulation and acoustic noise.^29^ A comparison between GRASE and TEMPURA could be carried out in future studies.

This study has several limitations. Firstly, the patient cohort consisted of 24 individuals with 29 tumors, all classified as low‐to‐intermediate risk (Gleason score 3 + 3 or 3 + 4). In addition, biopsy techniques are prone to sampling error and the median time from biopsy was 334.5 days. However, this is not unexpected in an active surveillance cohort, where surveillance biopsy is rarely performed in the absence of MRI change.^30^, ^31^ Subsequent research will explore TEMPURA‐LWI performance in larger patient cohorts spanning a broader Gleason score range. Secondly, the HR method can potentially produce results with clearer tissue depiction and more detailed anatomical structures, but the improvement has not been quantitatively assessed. Future studies could utilize prostatectomy specimens with histological reference boundaries to assess whether improved spatial resolution enhances tumor delineation accuracy. Lastly, further optimization of the echo combination strategy could be explored—for example, combining more echoes at longer TEs to improve robustness in the presence of substantial signal decay.

## 5 CONCLUSION

This study applies a high acceleration method, TEMPURA, for LWI, which enables a fast standard‐resolution acquisition in 2.8 min as well as a HR acquisition in 5.4 min. Compared to the standard acquisition in 8.3 min, both the Fast and HR methods showed a high correlation in LWI measurements and consistent diagnostic performance in detecting malignant lesions.

## Supporting information


**Table S1.** Number of tumors by Gleason Grade and PI‐RADS Scores.
**Table S2**. LWI measurements of malignant and benign tissues using different methods.
**Table S3**. AUCs for detecting prostate carcinoma with DeLong test.
