# Supplementary material for: Fast and High‐Resolution luminal water imaging for prostate cancer diagnosis
Source: Magn Reson Med. 2025 Jul 4;94(5):2150–7. doi: 10.1002/mrm.30628 (PMC12393181; doi:10.1002/mrm.30628)
Supplement: Supplementary file 1 — Table S1. Number of tumors by Gleason Grade and PI‐RADS Scores. Table S2. LWI measurements of malignant and benign tissues using different methods. Table S3. AUCs for detecting prostate carcinoma with DeLong test. [file MRM-94-2150-s001.docx]

**Table S1. Number of Tumours by Gleason Grade and PI-RADS Scores**

|  | Gleason score | | | PI-RADS scores | | |
| --- | --- | --- | --- | --- | --- | --- |
|  | 3+3 | 3+4 | 4+4 | 3 | 4 | 5 |
| TZ  (9 in total) | 7 | 2 | 0 | 1 | 4 | 4 |
| PZ  (19 in total) | 8 | 10 | 1 | 2 | 12 | 5 |
| CZ  (1 in total) | 0 | 1 | 0 | 0 | 1 | 0 |

| Table S2. LWI Measurements of Malignant and Benign Tissues Using Different Methods | | | | | | | | |
| --- | --- | --- | --- | --- | --- | --- | --- | --- |
|  | | | **T2_short_** | **T2_long_** | **gmT2** | **A_short_** | **A_long_** | **LWF** |
| PZ | **Benign**  **(mean±SD)** | **Std.** | 56±7 | 433±96 | 113±34 | 110±46 | 40±20 | **0.29±0.12** |
|  |  | **Fast** | 73±8 | 414±96 | 135±42 | 60±18 | 26±13 | **0.30±0.13** |
|  |  | **HR** | 74±10 | 422±88 | 142±51 | 59±29 | 25±11 | **0.32±0.14** |
|  | **Malignant**  **(mean±SD)** | **Std.** | 57±7 | 371±128 | 69±18 | 134±48 | 10±10 | **0.08±0.08** |
|  |  | **Fast** | 75±8 | 325±89 | 89±19 | 69±25 | 7±7 | **0.10±0.09** |
|  |  | **HR** | 66±7 | 349±102 | 79±19 | 80±30 | 7±5 | **0.08±0.07** |
|  | **Spearman *r***  **(vs. Std.)** | **Fast** | 0.60 | 0.84 | 0.93 | 0.77 | 0.93 | **0.97** |
|  |  | **HR** | 0.21^*^ (*P*=0.17) | 0.69 | 0.89 | 0.58 | 0.91 | **0.90** |
|  | **Bias**  **(vs. Std.)** | **Fast** | 17 | -31 | 21 | -57 | -9 | **0.014** |
|  |  | **HR** | 14 | -16 | 21 | -52 | -10 | **0.018** |
| TZ | **Benign**  **(mean±SD)** | **Std.** | 61±6 | 393±85 | 77±11 | 121±26 | 14±8 | **0.11±0.06** |
|  |  | **Fast** | 73±8 | 337±63 | 93±15 | 76±21 | 12±6 | **0.13±0.07** |
|  |  | **HR** | 68±7 | 352±53 | 89±17 | 74±20 | 11±6 | **0.14±0.06** |
|  | **Malignant**  **(mean±SD)** | **Std.** | 51±5 | 512±123 | 54±5 | 173±21 | 3±1 | **0.02±0.01** |
|  |  | **Fast** | 68±9 | 341±96 | 72±10 | 102±30 | 4±4 | **0.04±0.02** |
|  |  | **HR** | 58±3 | 382±55 | 61±4 | 95±21 | 3±1 | **0.03±0.02** |
|  | **Spearman *r***  **(vs. Std.)** | **Fast** | 0.55 | 0.46 | 0.84 | 0.64 | 0.82 | **0.91** |
|  |  | **HR** | 0.73 | 0.65 | 0.92 | 0.59 | 0.88 | **0.93** |
|  | **Bias**  **(vs. Std.)** | **Fast** | 14 | -86 | 17 | -52 | -1 | **0.024** |
|  |  | **HR** | 7 | -64 | 11 | -56 | -1 | **0.024** |

**^*^** denotes P>0.05

Std. = the standard method (Standard); Fast = the fast method; HR = the high-resolution method.

| Table S3. AUCs for Detecting Prostate Carcinoma with DeLong test | | | | | | | | |  |
| --- | --- | --- | --- | --- | --- | --- | --- | --- | --- |
|  |  |  | **T_2-short_** | **T_2-long_** | **gmT_2_** | **A_short_** | **A_long_** | **LWF** | |
| PZ | **AUC (95%CI)** | **Std.** | 0.58  (0.39–0.76) | 0.65  (0.47–0.82) | 0.90  (0.81–0.99) | 0.69  (0.53–0.85) | 0.93  (0.86–1) | **0.93**  **(0.85–1)** | |
|  |  | **Fast** | 0.59  (0.41–0.77) | 0.77  (0.62–0.91) | 0.88  (0.78–0.99) | 0.62  (0.45–0.8) | 0.93  (0.84–1) | **0.92**  **(0.83–1)** | |
|  |  | **HR** | 0.71  (0.55–0.86) | 0.73  (0.56–0.89) | 0.91  (0.83–1) | 0.73  (0.58–0.88) | 0.95  (0.90–1) | **0.95**  **(0.89–1)** | |
|  | **DeLong’s *p*-value** | **Fast** | 0.87 | 0.05 | 0.54 | 0.32 | 0.80 | **0.47** | |
|  |  | **HR** | 0.35 | 0.38 | 0.76 | 0.62 | 0.18 | **0.33** | |
| TZ | **AUC (95%CI)** | **Std.** | 0.89  (0.77–1) | 0.77  (0.58–0.96) | 0.97  (0.92–1) | 0.93  (0.84–1) | 0.97  (0.93–1) | **0.97**  **(0.93–1)** | |
|  |  | **Fast** | 0.65  (0.41–0.88) | 0.46  (0.21–0.71) | 0.88  (0.75–1) | 0.81  (0.66–0.96) | 0.88  (0.72–1) | **0.96**  **(0.89–1)** | |
|  |  | **HR** | 0.97  (0.92–1) | 0.66  (0.45–0.88) | 0.99  (0.97–1) | 0.78  (0.61–0.95) | 0.97  (0.93–1) | **0.99**  **(0.96–1)** | |
|  | **DeLong’s *p*-value** | **Fast** | 0.08 | 0.08 | 0.16 | 0.13 | 0.21 | **0.48** | |
|  |  | **HR** | 0.29 | 0.40 | 0.38 | 0.08 | 1 | **0.55** | |

Std. = the standard method (Standard); Fast = the fast method; HR = the high-resolution method.
